# Parkin regulates microglial NLRP3 and represses neurodegeneration in Parkinson's disease

**DOI:** 10.1111/acel.13834

**Published:** 2023-04-07

**Authors:** Yi‐Qun Yan, Ran Zheng, Yi Liu, Yang Ruan, Zhi‐Hao Lin, Nai‐Jia Xue, Ying Chen, Bao‐Rong Zhang, Jia‐Li Pu

**Affiliations:** ^1^ Department of Neurology, The Second Affiliated Hospital, College of Medicine Zhejiang University Hangzhou Zhejiang 310009 China

**Keywords:** microglia, neuroinflammation, NLRP3, Parkin, ubiquitination

## Abstract

Microglial hyperactivation of the NOD‐, LRR‐, and pyrin domain‐containing 3 (NLRP3) inflammasome contributes to the pathogenesis of Parkinson's disease (PD). Recently, neuronally expressed NLRP3 was demonstrated to be a Parkin polyubiquitination substrate and a driver of neurodegeneration in PD. However, the role of Parkin in NLRP3 inflammasome activation in microglia remains unclear. Thus, we aimed to investigate whether Parkin regulates NLRP3 in microglia. We investigated the role of Parkin in NLRP3 inflammasome activation through the overexpression of Parkin in BV2 microglial cells and knockout of Parkin in primary microglia after lipopolysaccharide (LPS) treatment. Immunoprecipitation experiments were conducted to quantify the ubiquitination levels of NLRP3 under various conditions and to assess the interaction between Parkin and NLRP3. In vivo experiments were conducted by administering intraperitoneal injections of LPS in wild‐type and Parkin knockout mice. The Rotarod test, pole test, and open field test were performed to evaluate motor functions. Immunofluorescence was performed for pathological detection of key proteins. Overexpression of Parkin mediated NLRP3 degradation via K48‐linked polyubiquitination in microglia. The loss of Parkin activity in LPS‐induced mice resulted in excessive microglial NLRP3 inflammasome assembly, facilitating motor impairment, and dopaminergic neuron loss in the substantia nigra. Accelerating Parkin‐induced NLRP3 degradation by administration of a heat shock protein (HSP90) inhibitor reduced the inflammatory response. Parkin regulates microglial NLRP3 inflammasome activation through polyubiquitination and alleviates neurodegeneration in PD. These results suggest that targeting Parkin‐mediated microglial NLRP3 inflammasome activity could be a potential therapeutic strategy for PD.

## Graphical abstract

Parkin regulates microglial NLRP3 inflammasome activation through polyubiquitination and alleviates neurodegeneration in PD. Combined genetic deletion of Parkin and stimulation by LPS created a PD phenotype that more accurately modeled that of humans. Targeting Parkin‐mediated microglial NLRP3 inflammasome activity using HSP90 inhibitor could be a potential therapeutic strategy for PD.

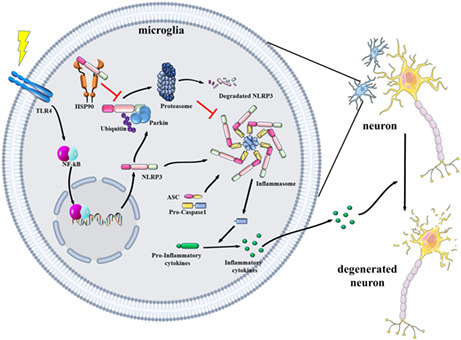

## 1 INTRODUCTION

Parkinson's disease (PD) is a common neurodegenerative disease characterized by progressive dopaminergic neuron loss (Balestrino & Schapira, 2020). PD pathogenesis has not been clearly elucidated; however, among the existing theories, the interplay between genetic susceptibility and environmental effects is widely accepted (Kalia & Lang, 2015). The *PRKN* gene is the most common autosomal recessive gene in PD; it encodes the Parkin protein, a RING‐type E3 ubiquitin ligase. Parkin has a recognized role in mitophagy and quality control in the mitochondria (Pickrell & Youle, 2015). However, Parkin‐deficient mice exhibit a different phenotype than that in humans, causing late‐onset PD (Noda et al., 2020; Paul & Pickrell, 2021), possibly because laboratory mice are typically housed in specific‐pathogen‐free environments. PTEN‐induced kinase 1 (PINK1) and Parkin work cooperatively in the mitophagy process. PINK1 phosphorylates ubiquitin to recruit and activate Parkin in damaged mitochondria (Kane et al., 2014; Koyano et al., 2014). Therefore, mice with PINK 1 deficiency mice have similar phenotypes as mice with Parkin deficiency mice (Paul & Pickrell, 2021). Matheoud et al. demonstrated that intestinal infection triggers motor symptoms in PINK1^−^ mice (Matheoud et al., 2019). Frank‐Cannon et al. reported that a low‐dose continuous injection of LPS induces neurodegeneration in Parkin‐deficient mice (Frank‐Cannon et al., 2008), revealing details about the interplay between Parkin deficiency and neuroinflammation.

Central and peripheral inflammation plays an important role in driving PD pathology (Nguyen & Palm, 2022; Pajares et al., 2020). Briefly, gut microbiota dysbiosis and immune system alterations have an influence on the central nervous system, leading to microglial activation, inflammatory response, and subsequent neuronal death (Lazdon et al., 2020; Sampson et al., 2016). Among the numerous inflammatory pathways, NOD‐, LRR‐ and pyrin domain‐containing 3 (NLRP3) inflammasome activation is a main source of inflammatory regulation in microglia (Haque et al., 2020). NLRP3 expression is induced after stimulation, after which the complex incorporates the apoptosis‐associated speck‐like protein containing a CARD (PYCARD/ASC) adaptor and the effector pro‐caspase‐1 into the inflammasome assembly and induces protein cleavage and cytokine secretion (Huang et al., 2021). Elevated NLRP3 activation was found in patients with PD and in various animal models of PD, driving dopaminergic neuron death. Inhibition of NLRP3 prevents PD pathology in mice, indicating a crucial role of the NLRP3 inflammasome in the onset of PD (Gordon et al., 2018; Lee et al., 2019; von Herrmann et al., 2018). Recent studies have shown that Parkin may exhibit broad substrate selectivity (Shires et al., 2017), and inflammatory pathways are possible targets of Parkin (Quinn et al., 2020; Sliter et al., 2018), although the underlying mechanism remains unclear.

Increasing evidence suggests that Parkin may regulate inflammation through the ubiquitination of NLRP3. NLRP3 is a protein with multiple ubiquitination sites, which induce NLRP3 degradation through autophagy (Han et al., 2019). Polyubiquitination of NLRP3 by tripartite motif containing 31 (TRIM31), another RING‐type E3 ubiquitin ligase, induces ubiquitin‐proteasome degradation (Song et al., 2016). De‐ubiquitination of NLRP3 facilitates its activation; conversely, ubiquitination of NLRP3 inhibits its activation (Juliana et al., 2012; Tang et al., 2021). Ubiquitination is a vital form of post‐translational modification, the status of which determines whether NLRP3 becomes activated or degraded. Recently, NLRP3 was reported to be a substrate of Parkin in neurons and BEAS‐2B epithelial cells (Ge et al., 2021; Panicker et al., 2022). However, no experimental evidence has shown whether this occurs in the microglia. The total RNA sequence obtained from Genevestigator software revealed significantly higher *PRKN* RNA expression levels in microglia than in other neural cells, indicating that Parkin may play an important role in microglia (Figure S5). However, whether Parkin can regulate microglial activation and whether this regulation occurs through NLRP3 inflammasome activity is unknown.

Here, we demonstrate that Parkin regulates NLRP3 activation via polyubiquitination in microglia. Moreover, Parkin deficiency exacerbates microglia activation and neurodegeneration in LPS‐induced PD mice, thus indicating that promoting Parkin activity may represent a strategy for inhibiting NLRP3‐related neuroinflammation to treat PD.

## 2 METHODS

### 2.1 Standard protocol approvals

Animal care and experiments were conducted in accordance with the National Institutes of Health Guide for the Care and Use of Laboratory Animals and with approval from the Institutional Animal Care and Use Committee of The Second Affiliated Hospital of Zhejiang University (Approval No. 55 of 2020).

### 2.2 Mice and study design

B6.129S4‐Prkn^tm1Shn^/J mice from The Jackson Laboratory were used as a Parkin KO animal model. Wild‐type C57BL6/J were obtained from Vital River Laboratory Animal Technology Co. Parkin KO mice were backcrossed with WT mice for more than four generations; homozygous mutant and WT offspring were used for experiments. Mouse genotypes were identified by PCR according to protocol provided by the Jackson Laboratory. Animals were housed in the Laboratory Animal Center of Zhejiang University on a 12‐h/12‐h light/dark cycle in a temperature‐controlled facility. For the short‐term inflammation model, 8–12 weeks old WT or Parkin KO mice with an isogenic background were used. Mice were divided into the following four groups (*n* = 6/group) depending on genotype and whether they were treated with LPS or phosphate‐buffered saline (PBS): (1) WT‐PBS, (2) WT‐LPS, (3) KO‐PBS, (4) KO‐LPS. Mice were administered intraperitoneal injections of LPS (5 mg/kg) or PBS at equal volumes. Animals were sacrificed the next day, with three animals used for protein extraction and three used for immunohistochemistry of sectioned brains from each group. For the long‐term inflammation model, 20–24 weeks old mice with an isogenic background were used. About 16 WT (eight males and eight females) and 16 Parkin KO (eight males and eight females) mice with isogenic backgrounds were divided into four groups (*n* = 8/group, with equal sex distributions). Animals underwent Rotarod training over 5 consecutive days and were then administered intraperitoneal injections of LPS (5 mg/kg) or PBS at equal volume. Behavioral tests were conducted as follows: open field test 1‐week post‐injection; Rotarod test 1‐, 2‐, 4‐, and 6‐months post‐injection; and pole test 6 months post‐injection. Two mice in the KO‐LPS group died after LPS injection, and one mouse in the KO‐PBS group died from fighting with other mice; they were excluded from the study.

### 2.3 Cell culture

Mouse primary microglia (PM) were isolated from WT and Parkin KO mice on a C57BL6/J background. Newborn mice (P0) were decapitated, and cortices, without the meninges or blood, were collected and digested in 0.25% trypsin at 37°C for 15 min. Cells from three cortices were filtrated through a 70‐μm‐pore‐size filter and seeded in one T‐75 culture flask coated with poly‐D‐lysine. The mixed glial culture was cultivated for 1 week, then subjected to vibration at 150 rpm for 1 h to collect PM. Mouse BV2 cells, human SH‐SY5Y cells, and human embryonic kidney 293 T cells were obtained from the Cell Bank of the Chinese Academy of Sciences. Cells were cultured in Dulbecco's modified Eagle's medium (DMEM, Gibco) supplemented with 10% fetal bovine serum (FBS) (Invitrogen‐Gibco) at 37°C under 5% CO_2_. Cells were starved overnight before the IP test to induce protein degradation. For the co‐incubation study, PM were treated with LPS for 4 h and then ATP for 30 min, and the supernatant was collected. Then the supernatant was added into SH‐SY5Y cell cultures for another 48‐h incubation. The reagent concentrations were as follows: LPS at 500 ng/mL for PM and 1 μg/mL for BV2 cells; adenosine triphosphate (ATP) at 2.5 μM for both; MG‐132 at 20 μM for 24 h; 3‐methyl adenine (3‐MA) at 2.5 mM for 24 h; 17‐AAG 1 μM for 24 h; MCC950 at 1 μM co‐stimulation with LPS; Cycloheximide (CHX) at 100 μg/mL was added into cell culture medium at 0, 4, 8, and 12 h after a 12‐h LPS treatment.

### 2.4 Chemicals and reagents

LPS (L2880), ATP (A6419), and CHX (C7698) were purchased from Sigma–Aldrich. MG‐132 (HY‐13259), 3‐MA (HY‐19312), 17‐AAG (HY‐10211), and MCC950 (CP‐456773) were obtained from MedChemExpress. Lipofectamine 3000 (L3000015) was purchased from Invitrogen mouse interleukin 1 beta (IL‐1β) enzyme‐linked immunosorbent assay (ELISA) kit (EK0394) was purchased from Boster Bio. Cell Counting Kit‐8 (CCK‐8) assay (40203) was obtained from Yeasen Biotechnology. Nissl Staining Solution (C0117) and Lipo293 (C0521) was purchased from Beyotime. IL‐1β (12242S, 1:800, WB), Parkin (4211S, 1:800, WB), ubiquitin (3936S, 1:2000, WB), p62 (5114 T, 1:800, WB), and phosphorylated nuclear factor kappa B (p‐NF‐kB) (3033S, 1:800, WB) antibodies were purchased from Cell Signaling Technology. Anti‐NLRP3 (used for WB, AG‐20B‐0014, 1:1000), anti‐caspase‐1 (AG‐20B‐0042, 1:800, WB), and anti‐ASC (AG‐25B‐0006, 1:1000, WB) antibodies were purchased from AdipoGen Life Sciences. Anti‐NLRP3 (used for Co‐IP, NBP2‐12446) was purchased from Novus Biologicals. Alexa Fluor 488‐conjugated anti‐rabbit (A‐11008, 1:500), Alexa Fluor 594‐conjugated anti‐mouse (A‐11032, 1:500), Alexa Fluor 488‐conjugate anti‐rat (A‐48262, 1:500), and anti‐CD11b (14–0112, 1:500, IF) antibodies were purchased from Invitrogen. Anti‐Iba1 (019–19,741, 1:500, IF) was obtained from FUJIFILM Wako Pure Chemical Corporation. Anti‐Bax (50599‐2‐IG, 1:2000, WB) was obtained from Proteintech. Anti‐FLAG (M1403, Co‐IP) and anti‐Parkin (ET1702, 1:300, IF) antibodies were obtained from HuaBio. Anti‐Myc (AE070, IP), anti‐K48 polyubiquitin (A18163, 1:2000, WB), anti‐Bcl‐2 (A19639, 1:1000, WB), and anti‐HSP90α (A5006, 1:500, IF) antibodies were purchased from ABclonal.

### 2.5 Co‐immunoprecipitation (Co‐IP)

Co‐IP was conducted according to a commercial instructions of Co‐IP kits (Thermo Scientific™, 88,804). Briefly, two 10 cm dishes of BV2 cells were transfected with flag‐Parkin plasmid for 48 h and then treated with LPS for 4 h. Cells from each 10 cm dish were harvested with 500 μL NP‐40 detergent containing protease inhibitors. Five percent of cell lysate was used for the input; the remainder was incubated overnight with magnetic beads coated with antibodies at 4°C. Cell lysate was incubated with 2 μg target antibody or IgG control. NLRP3 or Flag antibodies were used for Co‐IP and reverse Co‐IP. Beads were boiled at 100°C for 10 min in 20 μL loading buffer to elute protein.

### 2.6 Behavioral testing

For the open field test, mice were left undisturbed in the testing room for 30 min for adaptation, then placed in the middle of the field and allowed to roam freely for 5 min. The distance traveled was measured by SMART video tracking software (Smart 3.0). The field was equally divided into 16 areas; the sum of the distances travelled in all 16 areas indicated the total distance, and the sum of the distance travelled in the four middle areas represented the “middle distance.” The activity of rearing was recorded by an observer during the 5‐min experiment.

For the Rotarod test, mice were placed on a rotating rod accelerating from 4–40 rpm over a 5 min period. Before testing, mice were pre‐trained for 5 days, and the average fall latency was recorded as the baseline. In each experiment, mice were tested five separate times, and the average latency to fall was recorded. The cut‐off latency was 150 s. The Rotarod apparatus was provided by Panlab, Barcelona, Spain (LE8205).

For the pole test, a wooden instrument, 50‐cm in length and a pole with a 1‐cm diameter, was used. A ball with a 2.5‐cm diameter was situated at the top of the pole. During the test, the pole was raised at a 90° angle to the ground. Mice were placed on the ball and allowed to climb down spontaneously. The time taken to reach the bottom from the top was recorded as the climb time. Five independent tests were conducted for each mouse to determine the average.

### 2.7 Quantitative reverse transcription PCR (RT‐qPCR)

Total RNA was extracted with RNAiso Plus (Takara 9108) according to the manufacturer's instructions. 5× Prime Script RT master mix (Takara, RR036A) was used for reverse transcription. TB Green Premix Ex Taq II (Takara, RR420A) for qPCR was used with the StepOnePlus Real‐Time PCR System (Applied Biosystems). The following primer sequences were used for RT‐qPCR: 5’‐GCAACTGTTCCTGAACTCAACT‐3′, 5’‐ATCTTTTGGGGTCCGTCAACT‐3′ for IL‐1β; 5’‐GGCCCTTGCTTTCTCTTCG‐3′, 5′‐ATAATAAAGTTTTGATTATG T‐3′ for IL‐6; 5′‐ CCTGTAGCCCACGTCGTAG‐3′, 5′‐ GGGAGTAGACAAGGTACAACCC‐3′ for tumor necrosis factor alpha (TNF‐α); 5’‐ACAAGGCACGGGACCTATG‐3′, 5’‐TCCCAGTCAGTCCTGGAAATG‐3′ for caspase‐1; 5’‐TTGACACGAGTGGACCTGAG‐3′, 5’‐GACCTCTGGCTGCTTCTGAA‐3′ for Parkin; 5’‐ATTACCCGCCCGAGAAAGG‐3′, 5’‐TCGCAGCAAAGATCCACACAG‐3′ for NLRP3; 5’‐CGGGAGGGTAACCATAAGCC‐3′, 5’‐GTCTGCTTTGCTGTGATGCC‐3′ for arginase 1 (ARG‐1); and 5’‐CAGCTGGGCTGTACAAACCTT‐3′, 5’‐CATTGGAAGTGAAGCGTTTCG‐3′ for inducible nitric oxide synthase (iNOS).

### 2.8 Immunofluorescence

Mice were anesthetized with 1% pentobarbital through intraperitoneal injection. Anesthetized mice were perfused intracardially with PBS first and then 4% paraformaldehyde to fix brain tissue. Brains were fixed in 4% paraformaldehyde for 24 h then dehydrated and cryoprotected in 30% sucrose until tissues sunk to the bottom of the container. Frozen brains embedded in Optimal Cutting Temperature (OCT) compound (Sakura, 4583) were sliced on a cryostat microtome into 30‐μm coronal sections (for neuron counting) or 12‐μm coronal sections (for microglia and protein labeling).

Brain slices and fixed cell climbing slices were incubated in 5% bovine serum albumin (BSA) containing 0.3% Triton X‐100 for 1 h, followed by an overnight primary antibody incubation at 4°C. The next day, slices were washed three times in PBS and incubated in a secondary antibody solution for 1.5 h at 37°C. Subsequently, slices were washed five times and mounted with anti‐fade agents containing 4′, 6‐diamidino‐2‐phenylindole (DAPI). Images were captured using a fluorescence microscope (Leica DM6B) and confocal laser endomicroscopy (Olympus FV1200). The number of tyrosine hydroxylase‐positive (TH^+^) neurons were counted via stereology. Thirty‐μm coronal sections of midbrain tissue containing substantia nigra pars compacta (SNc) were used for neuronal counting. We examined 6–7 sections per brain at similar layer and calculated the average number of cells.

### 2.9 Western blotting (WB)

Cell lysates were collected using radioimmunoprecipitation assay (RIPA) buffer containing a protease inhibitor cocktail and phosphatase inhibitor mixture and then submitted to ultrasonification for 5 s. Brains were homogenized using a tissue grinder in RIPA buffer containing a protease inhibitor cocktail. Cell lysates or tissue homogenates were then centrifuged at 13,800 *g* at 4°C for 15 min, and the supernatant was boiled in 1x loading buffer for 8 min at 100°C. WB was conducted as previously described (Zheng et al., 2021).

### 2.10 Mitochondrial reactive oxygen species (ROS) detection

Mitochondrial ROS were measured using a mitochondrial superoxide indicator (MitoSOX) (mtSOX Deep Red, DoJinDo Laboratories). MitoSOX was diluted to a 10 μmol/L working solution in DMEM, then added to cell culture plates and incubated for 30 min. PM were washed twice with PBS, then digested in trypsin at 37°C for 15 min. ROS levels were quantified by measuring fluorescence intensity using a flow cytometer (CytoFLEXLX, Beckman). Data processing was conducted using CytExpert Software (V2.0).

### 2.11 Statistical analysis

WB and immunofluorescence images were quantized using FIJI ImageJ software based on pixel intensity (https://imagej.net/software/fiji). Co‐localization analysis and calculation of Pearson's R values were conducted using the coloc‐2 plugin. Raw data process was conducted using Microsoft Excel 2013. Statistical analysis was conducted using GraphPad Prism 8.0 software. Student's *t*‐tests, one‐way analysis of variance (ANOVA), and two‐way ANOVA were used for significance testing. Tukey's multiple comparison post hoc test was used for comparisons between groups when appropriate.

## 3 RESULTS

### 3.1 Parkin regulates NLRP3 inflammasome activation in BV2 cells and PM

BV2 cells were stimulated with LPS and ATP, then NLRP3 inflammasome activation was confirmed by a significant increase in NLRP3 expression and IL‐1β secretion. Parkin protein levels did not decrease until 24 h post‐stimulation (pS), and they were dramatically downregulated at 48 h pS (Figure S1a,b). Significant decreases in Parkin expression were observed at the mRNA level beginning 4 h after LPS stimulation; this change was matched by elevated mRNA levels of inflammatory cytokines (Figure S1b,c). The ELISA for Il‐1β in the supernatant confirmed the inflammasome activation at 4 h pS (Figure S1d). The expression of NLRP3 receded after 48 h. These results suggest that Parkin may involve in NLRP3 inflammasome activation. We then manipulated Parkin expression to determine its influence on NLRP3 activation and inflammation.

**FIGURE 1:**
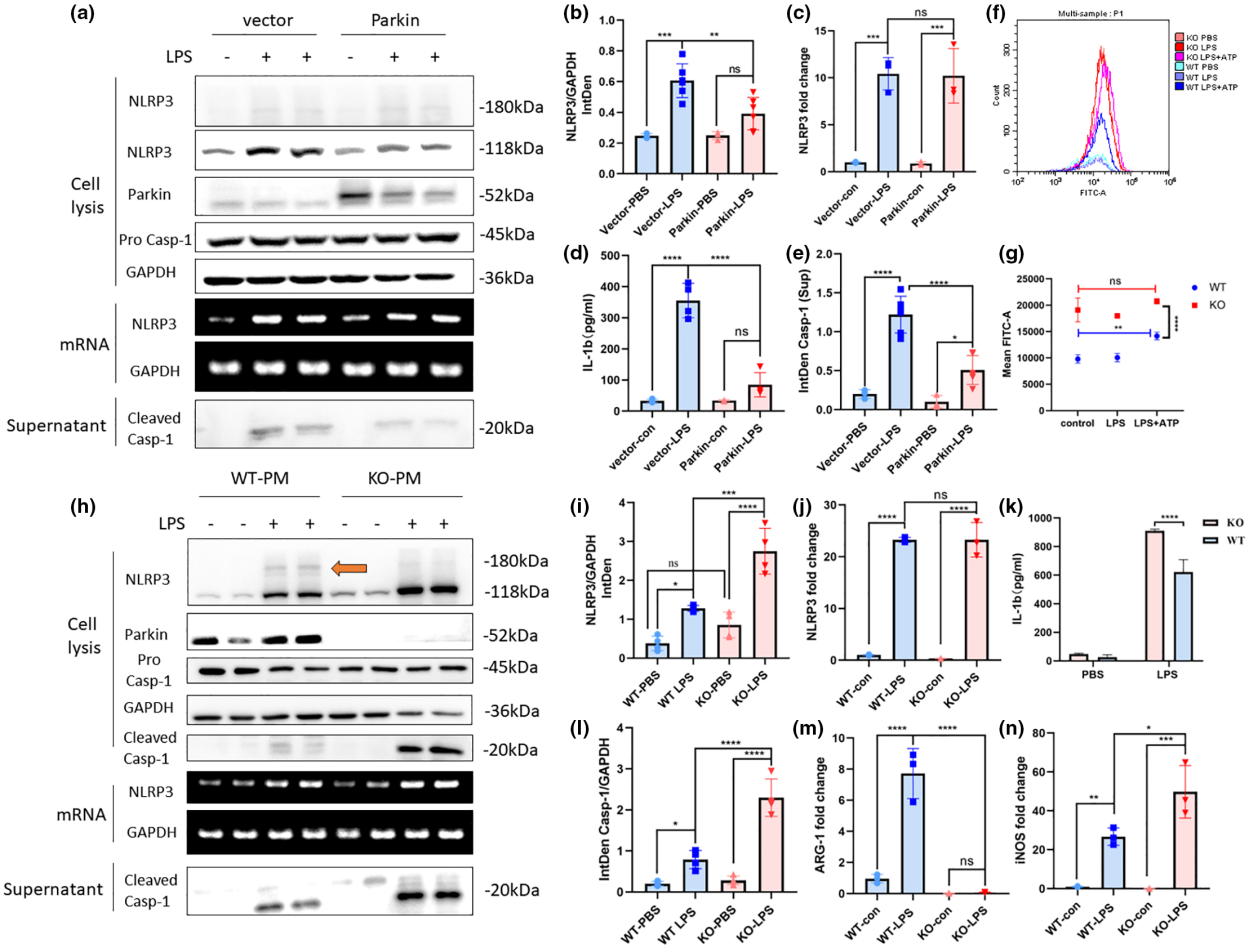
Parkin regulates NLRP3 inflammasome activation. (a) Overexpression of Parkin in BV2 cells alleviates NLRP3 inflammasome activation and the downstream inflammatory response but does not change the NLRP3 mRNA level. (b, i) Statistical analysis of the NLRP3 integrated density of WB experiments in BV2 cells (b) and PM (i). (d, k) ELISA of IL‐1β in the supernatant of BV2 cells (d) and PM (k). (c, j) qPCR of NLRP3 in BV2 (c) and PM (j). (e) Statistical analysis of cleaved Caspase‐1levels in supernatant of BV2. (f) Mitochondrial ROS levels of WT and Parkin KO PM quantified by flow cytometric analysis. (g) Statistical analysis of mean FTIC‐A in flow cytometric experiments. (h) Parkin KO exacerbates NLRP3 inflammasome activation and the downstream inflammatory response but does not alter the NLRP3 mRNA level. (l) Statistical analysis of the cleaved Caspase‐1 integrated density of WB experiments in PM. (m, n) qPCR of ARG‐1, and iNOS in PM. The orange arrow points out the post‐translational band of NLRP3. NLRP3, NOD‐, LRR‐, and pyrin domain‐containing 3; WB, Western blot; PM, primary microglia; ELISA, enzyme‐linked immunosorbent assay; IL‐1β, interleukin 1 beta; qPCR, quantitative polymerase chain reaction; ROS, reactive oxygen species; WT, wild‐type; KO, knockout; ARG‐1, arginase 1; iNOS, inducible nitric oxide synthase; ns, not significant. *p < 0.05, **p < 0.01, ***p < 0.001, ****p < 0.0001.

Plasmids overexpressing Parkin were constructed and transfected into BV2 cells for 48 h, followed by stimulation with LPS. A FLAG‐tagged plasmid served as a transfection control. There was a significant decrease in NLRP3 protein expression in BV2 Parkin‐overexpressing cells compared with that of the control group after LPS stimulation, whereas no difference in NLRP3 mRNA levels was observed (Figure 1a–c). Levels of cleaved caspase‐1 and IL‐1β (assessed by WB and ELISA, respectively) in the supernatant were also decreased in Parkin‐overexpressing cells (Figure 1a,d,e).

PM from Parkin KO and WT mice were used to detect the influence of Parkin deficiency on NLRP3 inflammasome levels. After LPS stimulation, NLRP3 levels were significantly upregulated in PM from Parkin KO mice, although the mRNA levels did not differ between PM from WT and KO mice (Figure 1h–j). Translocation of NF‐kB into the nucleus was similar, regardless of Parkin expression levels in microglia (Figure S1g,k). NLRP3 was not significantly upregulated in PM of KO mice without LPS treatment. Notably, an ~180 kDa band was clearly visible in PM of the WT mice after LPS treatment; this was not the case in the PM of the KO group (Figure 1h). This band was also seen in the BV2 cells, although it was fainter. In the pathways downstream of NLRP3 activation, cleaved caspase‐1 levels in cell lysates and culture supernatants were also markedly upregulated in the Parkin KO group. Levels of secreted IL‐1β in the supernatant were also significantly higher in the Parkin KO group (Figure 1k). There were opposite changes in expression of the M1 marker iNOS and the M2 marker ARG‐1 in PM of the WT and Parkin KO mice. iNOS transcription was slightly elevated, whereas ARG‐1 expression dramatically decreased in Parkin KO microglia following inflammatory stimulation, relative to the levels in WT microglia (Figure 1m,n). iNOS and ASC protein levels were increased in PM of the KO group (Figure S1h).

ROS are another important trigger of NLRP3 inflammasome activation, and Parkin deficiency may cause overexpression of mitochondrial ROS. Quantification of the fluorescence intensity of MitoSOX staining revealed elevated mitochondrial ROS level in PM of the Parkin KO group compared to levels in WT microglia, although no difference was seen between the microglia of the KO mice treated with PBS, LPS, or LPS + ATP (Figure 1f,g). Integration of the mitochondrial ROS results with the levels of NLRP3 mRNA and NF‐kB in PM of KO mice suggested that ROS have a limited effect on NLRP3 expression.

### 3.2 Interaction between Parkin and NLRP3 after LPS stimulation

**FIGURE 2:**
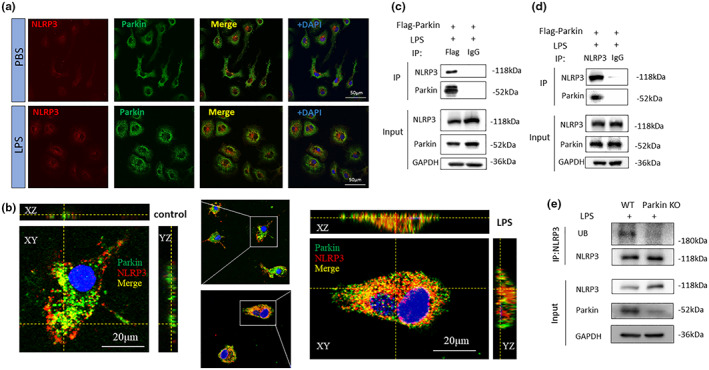
Interaction between NLRP3 and Parkin. (a) Co‐localization of NLRP3 with Parkin assessed via confocal microscopy. (b) 3D‐reconstructed image of NLRP3 and Parkin in BV2 cells via two‐photon microscopy. (c, d) Co‐immunoprecipitation experiment conducted on BV2 cells using a FLAG‐Parkin antibody and an NLRP3 antibody. (e) Immunoprecipitation conducted on WT or Parkin KO primary microglia using NLRP3 antibody. Ubiquitin and NLRP3 was detected. NLRP3, NOD‐, LRR‐ and pyrin domain‐containing 3; WT, wield type; KO, knockout.

To determine whether the ~180 kDa band of NLRP3 was a posttranslational modification band mediated by Parkin, we tested whether Parkin and NLRP3 interacted. We performed protein–protein affinity prediction using the PPA‐Pred2 website (www.iitm.ac.in/bioinfo/PPA_Pred/prediction.html). The predicted value of Delta G (binding free energy) was −12.73 kcal/mol, and the Kd (dissociation constant) was 4.59e‐10 M. Confocal immunofluorescence microscopy of LPS‐treated PM revealed Parkin and NLRP3 co‐localization (Figure 2a). Two‐photon fluorescence imaging was also used for BV2 cells after LPS treatment. The 3D‐reconstructed image revealed colocalization between Parkin and NLRP3 (Figure 2b). BV2 cells over‐expressing FLAG‐Parkin were used for Co‐IP experiments and LPS stimulation was used for NLRP3 overexpression. Magnetic beads were coated with Flag antibody or IgG control. A Co‐IP band of NLRP3 was seen in the anti‐Flag group but not in IgG group. Reverse experiments using anti‐NLRP3 and IgG control coated beads also showed a clear Parkin band in anti‐NLRP3 group but not in IgG group. Results revealed an interaction between NLRP3 and Parkin. Both experiments were repeated twice, and similar results were observed (Figure 2c,d). IP of NLRP3 from WT and Parkin KO PM triggered with LPS showed a ~ 180 kDa ubiquitin band in WT group and not in KO group (Figure 2e).

### 3.3 Parkin promotes K48‐linked polyubiquitination and NLRP3 degradation

**FIGURE 3:**
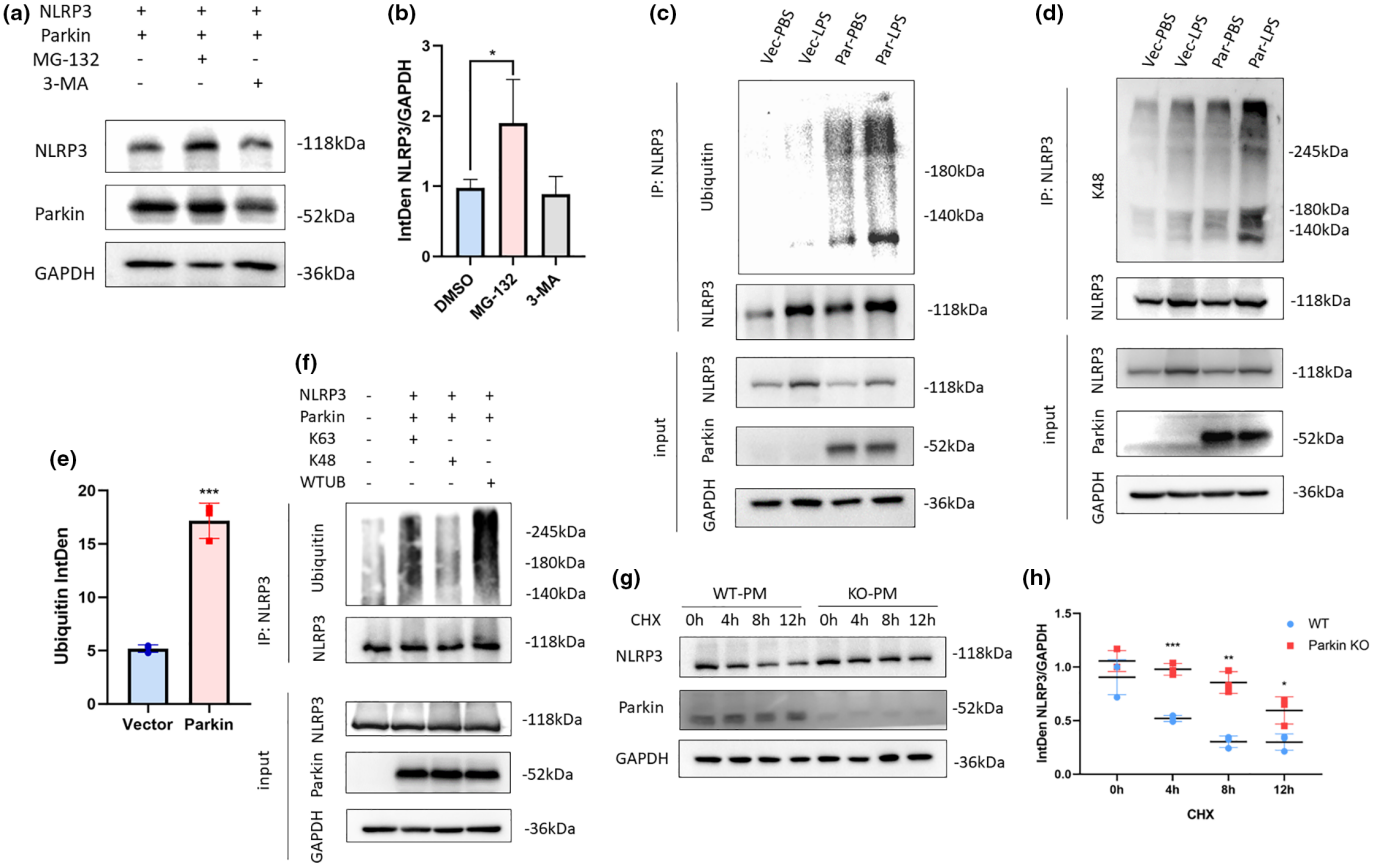
(a, b) Influence of two inhibitors, MG‐132, and 3‐MA on 293 T cells transfected with a Parkin and NLRP3 expression plasmid (a). Statistical analysis of NLRP3 levels in MG‐132, 3‐MA groups (b). (c–e) IP of NLRP3 in BV2 cells. Ubiquitination levels measured using a ubiquitin antibody (c) and a K48‐linked ubiquitin antibody (d). Statistical analysis of anti‐ubiquitin bands (e). (f) IP of NLRP3 in 293 T cells transfected with a different ubiquitin plasmid. (g, h) cycloheximide chase experiment on WT and Parkin KO primary microglia. Degradation speed was faster in WT group than in Parkin KO group (g), Statistical analysis showed difference at 4, 8, and 12 h time points (h). 3‐MA, 3‐methyl adenine; IP, immunoprecipitation; KO, knockout; NLRP3, NOD‐, LRR‐ and pyrin domain‐containing 3; WT, wild type. *p < 0.05, **p < 0.01, ***p < 0.001.

NLRP3 contains multiple ubiquitination sites and can undergo degradation via the ubquitin–proteasome pathway (UPP) (Song et al., 2016). Therefore, we investigated whether Parkin could mediate NLRP3 degradation through polyubiquitination. Protein can be degraded through the ubiquitin‐proteasome system or the autophagy‐lysosome system (Pajares et al., 2020). The proteasome inhibitor MG‐132 and the autophagy inhibitor 3‐MA were used to find out which way NLRP3 is degraded (Wu et al., 2021). MG‐132 could influence NLRP3 transcription in microglia in preliminary experiment (Figure S1j,k), so we exogenously expressed Parkin and NLRP3 in a 293 T cell line, followed by treatment with either MG‐132 or 3‐MA under starvation conditions. NLRP3 levels increased remarkably after MG‐132 treatment but were not elevated after 3‐MA treatment (Figure 3a,b). To further investigate NLRP3 degradation with or without the existence of Parkin, we conducted a cycloheximide chase experiment on WT and Parkin KO PM. Parkin deficiency significantly slowed down NLRP3 degradation at 4, 8, and 12 h after CHX treatment, indicating that Parkin promotes NLRP3 degradation in microglia (Figure 3g,h). Next, BV2 cells were transfected with Parkin expression plasmid or vector plasmid, followed by treatment with LPS or PBS. NLRP3 IP experiments revealed a significant upregulation in ubiquitination under Parkin overexpression, and the ubiquitination band was distinct after LPS treatment (Figure 3c,e).

One study suggested that K48‐linked ubiquitination mediated UPP degradation, whereas K63‐linked ubiquitination mediated non‐UPP modifications (Martínez‐Férriz et al., 2021). Here, K48‐linked polyubiquitination was assessed using a K48 linkage‐specific antibody and was significantly upregulated in BV2 cells with LPS‐induced Parkin overexpression (Figure 3d). An HA‐ubiquitin plasmid and HA‐ubiquitin plasmids with arginine substitutions of lysine at position 48 or 63 were co‐transfected with a Parkin and NLRP3 expression plasmid into 293 T cells. NLRP3 ubiquitination levels were significantly restrained when ubiquitin was mutated at position 48 but not at position 63 (Figure 3f).

### 3.4 Parkin deficiency induces greater microglial activation and neuroinflammation in mice after acute LPS treatment

Intraperitoneal injection of LPS could induce systemic acute inflammatory response (Azambuja et al., 2022). We observed mice activity on the next day after injection. Mice suffered from severe sickness and would not move in the cage. Mice in the short‐term inflammation model group were sacrificed immediately on the first day post injection for WB and immunofluorescence testing. Mice in the long‐term inflammation model group were subjected to the open field test and weighted to assess recovery condition.

**FIGURE 4:**
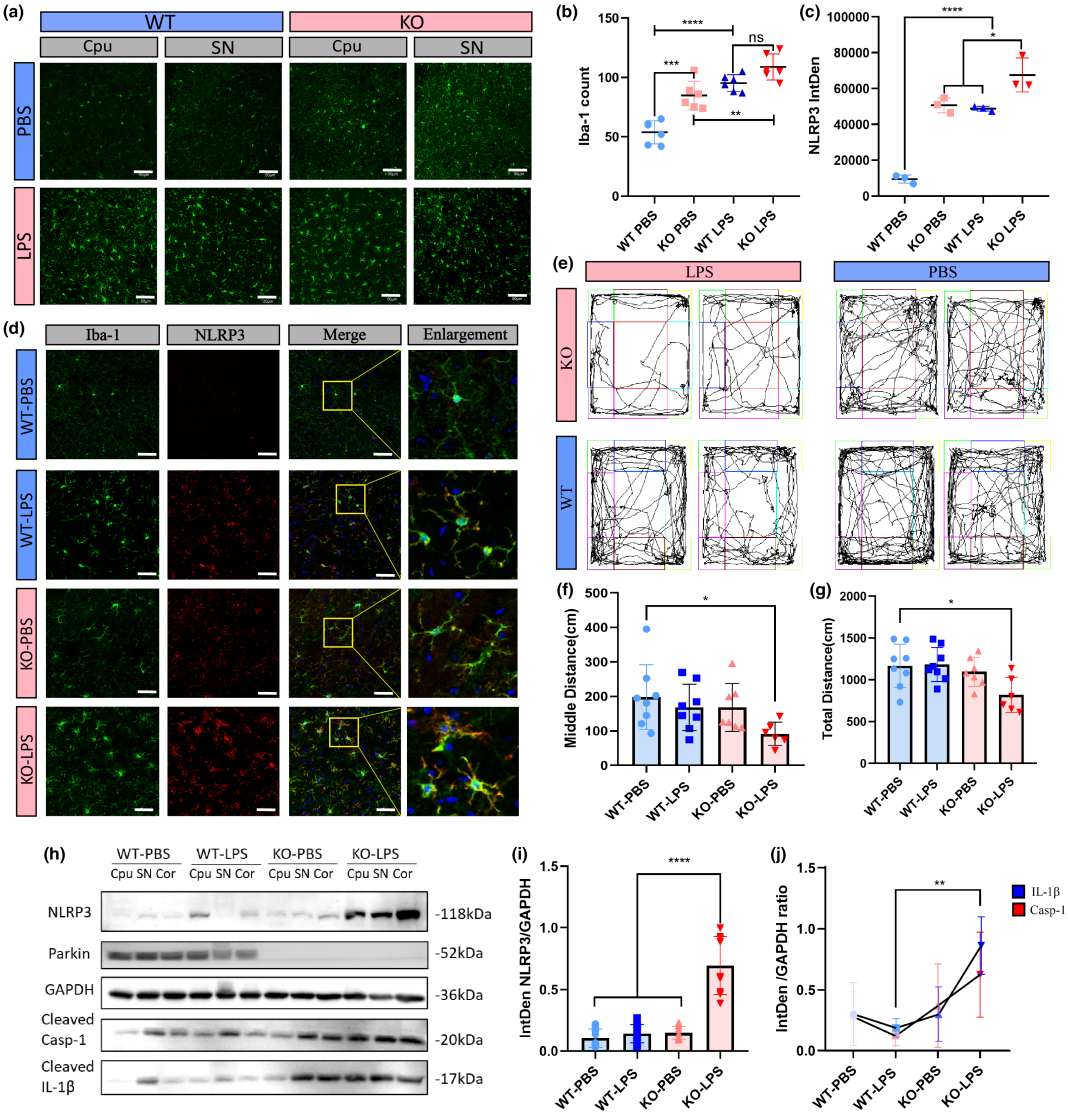
Parkin deficiency induced stronger microglia activation and heavier inflammatory response in mice. 12 WT and 12 Parkin KO male mice were divided into four groups (*n* = 6): WT‐PBS, WT‐ LPS, KO‐PBS, and KO‐LPS. In each group, three were used for immunofluorescence and three were used for WB. (a, b) Iba‐1 labeling of brain sections from SNc and striatum regions and Iba‐1‐positive cell counts. Each dot represents the SNc or striatum region of one mouse. (c, d) NLRP3 and Iba‐1 co‐labeling of brain sections of mice and statistical analysis of the integrated density of NLRP3 levels. Each dot represents one mouse. (e–g) Motion trajectories of mice (e) and distance traveled in the whole open field (f) or the middle area of the open field apparatus (g). (h–j) WB of NLRP3 inflammatory proteins in the brains of mice. Scale bar: 50 μm (a), 30 μm (d). KO, knock; LPS, lipopolysaccharide; NLRP3, NOD‐, LRR‐, and pyrin domain‐containing 3; SNc, substantia nigra pars compacta; WB, Western blot; WT, wield type. *p < 0.05, **p < 0.01, ***p < 0.001, ****p < 0.0001.

Iba‐1 fluorescent labeling was conducted to evaluate microglial activation in brain slices at post‐injection day 1. Confocal imaging showed significant microglial activation, characterized by larger cell sizes and amoeboid morphology, after intraperitoneal LPS injections in WT and KO mice. The differences were seen in the corpus striatum and substantia nigra (SN) areas (Figure 4a). In the KO‐PBS group, microglial activation was also evident in the absence of inflammatory stimulation (Figure 4a). Iba‐1‐positive cells were counted to evaluate the degree of microglial proliferation in response to different inflammatory triggers. The KO‐PBS group exhibited a significantly higher number of microglia than the WT‐PBS group. The same trend was found between the KO‐LPS and WT‐LPS groups, but the difference was not significant (Figure 4b).

NLRP3 levels were measured from confocal images. As expected, the mean NLRP3 fluorescence intensity was five times higher in the KO‐PBS group than the WT‐PBS group. NLRP3 levels were significantly upregulated in the KO‐LPS group compared to those in the WT‐LPS group (Figure 4c). Co‐labeling of NLRP3 and Iba‐1 revealed that the NLRP3 inflammasome was mainly activated in microglia, although some signals did not colocalize with microglial markers (Figure 4d). WB analyses showed significantly increased NLRP3 expression throughout the brain and elevated cleaved caspase‐1 and IL‐1β levels in downstream pathways (Figure 4h‐j).

On post‐injection day 7, open field testing indicated a greater systemic inflammatory response and longer recovery time in Parkin KO mice treated with LPS (Figure 4e). WT mice had almost recovered from LPS‐induced acute inflammation, with no difference in the total distance traveled in the whole field or middle field. However, Parkin KO mice were less active, with decreased travel distances in the whole field and center square (Figure 4f,g). Rearing activities were also decreased (Figure S2a). These results were indicative of sickness behavior and anxiety‐like symptoms in the KO‐LPS group on post‐injection day 7. Both WT and Parkin KO mice suffered from weight loss after LPS injection, although it was not significant in the WT group. Weight loss was higher in the Parkin KO group on post‐injection day 7 (Figure S2b).

### 3.5 Parkin deficiency induces long‐term microglial activation, chronic neuroinflammation, and greater dopaminergic neuron loss 6 months after LPS treatment

**FIGURE 5:**
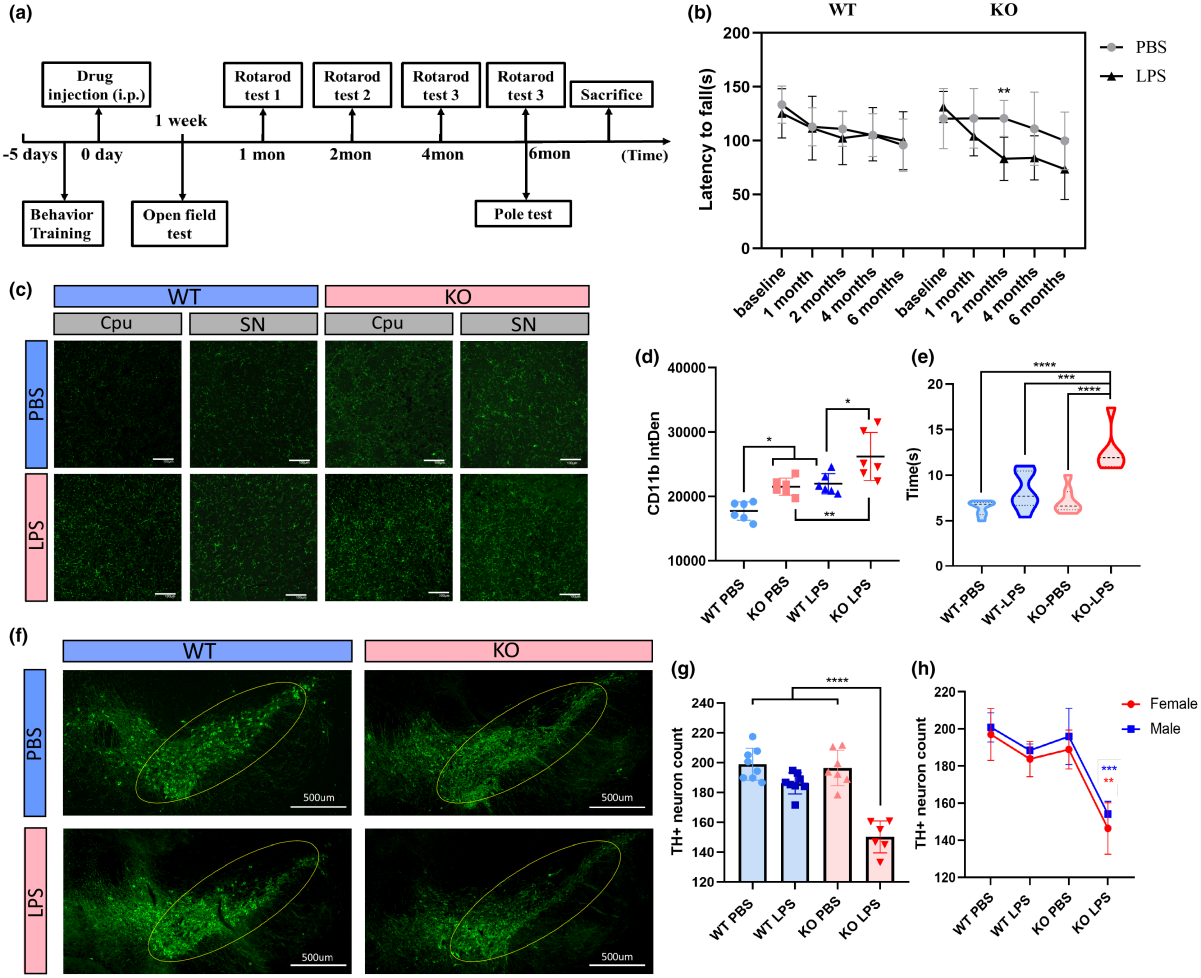
Neuroinflammation and PD‐related pathology 6 months after LPS treatment. 16 WT (8 males and 8 females) and 16 Parkin KO (8 males and 8 females) mice were divided into four groups (equal sex distribution): WT‐PBS, WT‐ LPS, KO‐PBS, KO‐LPS. Two in KO‐LPS group and one in KO‐PBS group died during the experiment and were excluded. (a) Schematic representation illustrating the experimental design (timeline). (b) Latency to fall in the Rotarod test at 6 months. (c, d) CD11b staining of brain sections of mice and integrated density of CD11b levels. Each dot represents the SNc or striatum region of one mouse. Scale bar: 100 μm. (e) Time required to reach the bottom of the pole from the top at 6 months post‐LPS injection. (f–h) TH staining in the SNc of the brains of mice. The yellow circle indicates the SNc. Quantification of the relative number of TH‐positive cells in the SNc, as determined for the whole group (g) and separately for each sex (h), although the results are the same. Each dot represents a mouse. Scale bar: 100 μm (c), 500 μm (f). KO, knockout; LPS, lipopolysaccharide; PD, Parkinson''s disease; SNc, substantia nigra pars compacta; TH, tyrosine hydroxylase; WT, wield type. *p < 0.05, **p < 0.01, ***p < 0.001, ****p < 0.0001.

To determine the long‐term effect of LPS treatment in Parkin KO mice, we conducted behavioral testing and pathological analyses. Rotarod testing was conducted at baseline and 1, 2, 4, and 6 months after intraperitoneal injection (Figure 5a). All four groups exhibited a decreased fall latency over this period due to aging and weight gain. No difference was seen between the WT‐LPS and WT‐PBS groups, whereas the KO‐LPS group performed worse than the KO‐PBS group beginning in the second month; however, the results at 4 and 6 months did not significantly differ due to large fluctuations (Figure 5b). Considering sex may influence behavior, we analyzed the Rotarod results of male and female mice separately and found a significant decrease in fall latency at month 2 in females and month 3 in males (Figure S2c,d). The pole test conducted 6 months post‐injection also revealed a significantly longer descent time in the KO‐LPS group (Figure 5e), indicating dysfunctional motor coordination.

The mean CD11b fluorescence intensity was distinctly higher in the Parkin KO‐LPS and KO‐PBS groups, indicating chronic microglial activation (Figure 5c). Co‐immunolabeling of CD11b and NLRP3 showed significant hyperexpression of NLRP3 in the SN, mostly within microglia (Figure S2e,f). Correspondingly, the numbers of TH^+^ neurons were significantly decreased in the KO‐LPS group, but not in the WT‐LPS group, which is consistent with the previous low‐dose LPS exposure model (Frank‐Cannon et al., 2008). The loss of TH^+^ neurons did not differ between male and female mice (Figure 5f–h). Nissl staining was also conducted to confirm neuron loss (Figure S3a,b). No significant difference of TH staining was seen in the corpus striatum (Figure S3c). Microglial inflammation, TH^+^ neuron loss, and the behavioral testing results were corroborative.

To investigate whether the neuronal death was caused by NLRP3 activation, we conducted an in vitro experiment by co‐culturing primary microglia and SH‐SY5Y cells. Parkin KO PM was treated with either LPS for 4 h followed by ATP for 30 min or MCC950 for 4 h together with LPS before ATP treatment. The supernatant was collected and added into SH‐SY5Y cells and incubated for 48 h. CCK‐8 assay did not show a significant cell death in different groups (Figure S3d); however, a significant decrease of Bcl‐2/Bax was observed in LPS group, and such decrease was rescued by NLRP3 inhibitor MCC950 (Figure S3e,f), indicating that activation of NLRP3 inflammasome in microglia leads to apoptotic execution propensity in neurons.

### 3.6 HSP90α is a potential regulator of Parkin‐induced NLRP3 degradation

Molecular chaperones are vital regulators of protein degradation (Margulis et al., 2020). Among all kinds of molecular chaperones, HSP90 was reported to inhibit NLRP3 inflammasome activation (Nizami et al., 2021). We performed protein–protein interaction (PPI) network analysis and constructed the PPI network using the STRING database (https://cn.string‐db.org/) (Figure S4a). The molecular chaperone protein, HSP90α, was a potential mediator between NLRP3 and Parkin. Furthermore, confocal fluorescence analysis of PM revealed moderate co‐localization between HSP90α and NLRP3 (Figure S4b). The co‐IP results also showed an interaction between HSP90α and both Parkin and NLRP3 (Figure S4c,d).

To explore the regulatory role of HSP90α in Parkin‐mediated NLRP3 degeneration, we used a specific inhibitor of HSP90, 17‐AAG. NLRP3 content decreased in microglia under 17‐AAG treatment, accompanied by a decreased downstream inflammatory response (Figure S4e). 293 T cells overexpressing NLRP3 and Parkin also exhibited increased NLRP3 degradation after 17‐AAG treatment (Figure S4f,g). IP of NLRP3 was conducted after 4‐h incubation with 17‐AAG, and NLRP3 levels were slightly decreased in the input sample, accompanied by a significant elevation of the ubiquitination band (Figure S4h,i). Therefore, HSP90α inhibition facilitates NLRP3 degradation through the UPP via Parkin mediation.

## 4 DISCUSSION

**FIGURE 6:**
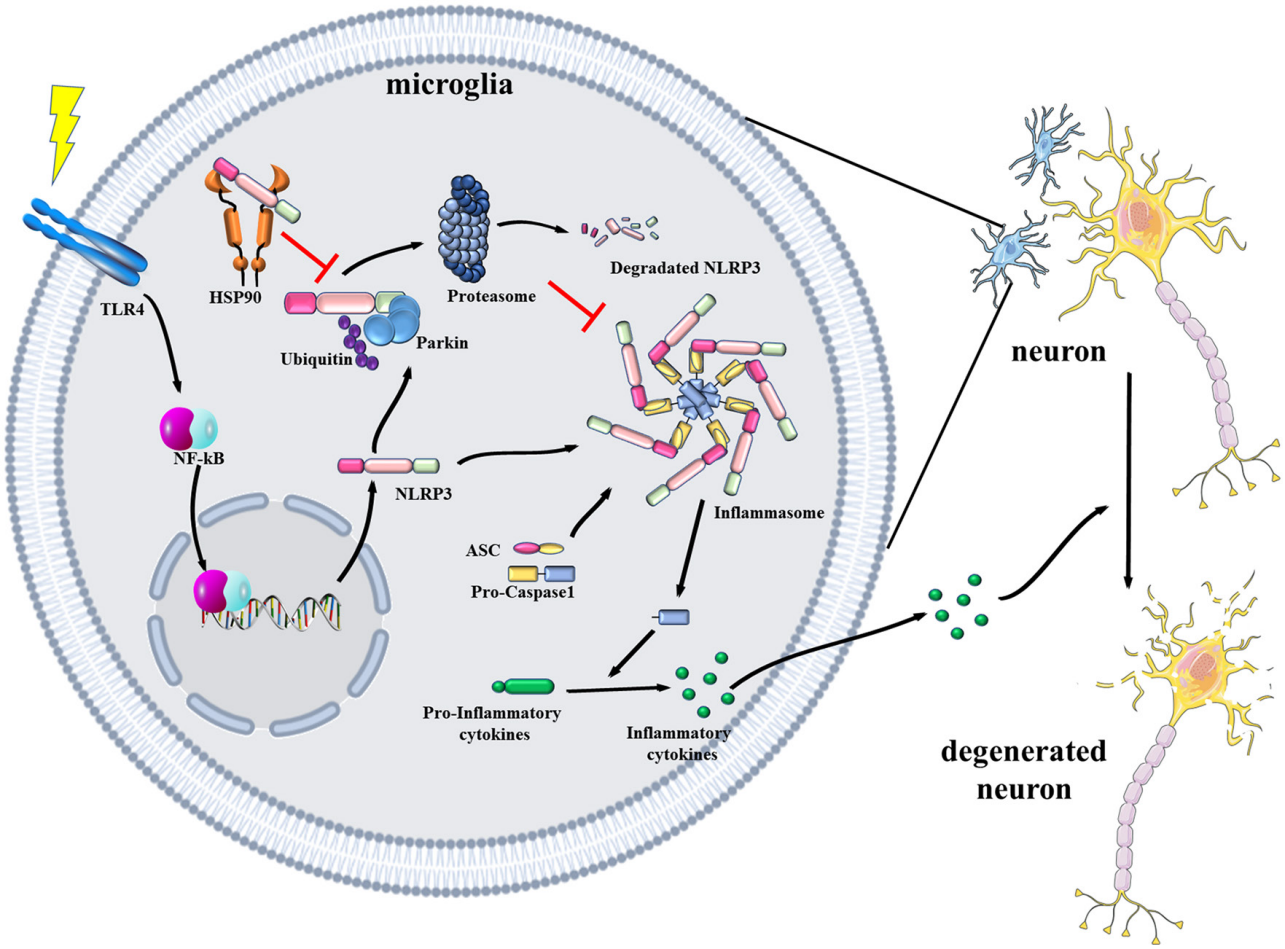
Schematic diagram of the regulatory mechanism through which Parkin and HSP90 modulate NLRP3‐associated inflammation. Microglia receive stimulation through the activation of surface receptors and then express NLRP3 and pro‐inflammatory proteins. NLRP3 can either assemble into an inflammasome or degrade through the ubiquitin‐proteasome pathway. Parkin is responsible for NLRP3 degradation by mediating K48‐linked polyubiquitination. The combination of NLRP3 with HSP90 could prevent NLRP3 from becoming degraded. NLRP3 inflammasome activation in microglia could create a cytotoxic environment for neurons and lead to neurodegeneration in Parkinson's disease. HSP90, heat shock protein 90; NLRP3, NOD‐, LRR‐ and pyrin domain‐containing 3.

This study confirmed that Parkin promotes NLRP3 degradation through K48‐linked polyubiquitination. Parkin deficiency exacerbates microglial NLRP3 inflammasome hyperactivation, facilitating motor dysfunction and dopaminergic neuron loss in LPS‐treated mice (Figure 6).

Intraperitoneal injection of LPS is a classical peripheral inflammation model that is also used as a chronic model of PD. Both single‐factor models, Parkin KO or LPS injection, require a long period to trigger the onset of PD. A previous study assessing a chronic inflammation model on Parkin KO mice showed similar neurodegeneration to our experiments, consistently proving that Parkin regulates inflammation (Frank‐Cannon et al., 2008). Our research reveals that a single high‐dose of LPS results in long lasting neuroinflammation in Parkin knockout mouse, suggesting that the combination of acute inflammatory response and gene mutation could be a trigger of late‐life neurodegeneration. These results support the two‐hit theory of PD pathogenesis, which involves synergy between gene mutations and environmental stress, and may support the gut‐brain axis theory (Avagliano et al., 2022; Gao et al., 2011). However, intraperitoneal injection of LPS causes acute systemic inflammation, making it an inappropriate means of mimicking mild inflammatory conditions such as gut microbiota dysbiosis. Further studies should explore whether aging induced mild systemic inflammation can accelerate the neurodegeneration observed in the Parkin deficiency mouse model.

Previous research demonstrated that NLRP3 acts as a Parkin substrate in neurons. Our results confirmed this mechanism also occurs in microglia and that Parkin induces NLRP3 degradation through the UPP, which is mediated by K48‐linked polyubiquitination. Ubiquitination is a main regulatory mechanism of NLRP3 activation. Ubiquitination of NLRP3 by a range of E3 ubiquitin ligases, including tripartite motif containing 65 (TRIM65), and Ariadne homolog 2 (ARIH2) inhibits inflammasome activation (Kawashima et al., 2017; Tang et al., 2021). However, other E3 ubiquitin ligases such as Pellino2, TNF receptor‐associated factor 6 (TRAF6), and HUWE1 were reported to facilitate NLRP3 priming (Guo et al., 2020; Humphries et al., 2018; Xing et al., 2017). These results uncover the complexity of NLRP3 ubiquitination and should be considered in future research. Our results showed that Parkin KO induces a significant shift in microglial activation markers ARG‐1 and iNOS, indicating that Parkin deficiency promotes microglial neurotoxicity. Moreover, microglial activation drives neuronal death in multiple neurodegenerative diseases, and drugs that target neuroinflammation and regulate the microglial activation state could effectively relieve symptoms and pathology. Recent research involving small molecule drug‐targeting of ubiquitin‐specific protease 7 showed that inhibiting deubiquitination in microglia could alleviate inflammation and attenuate PD pathogenesis (Zhang et al., 2022). Collectively, the findings suggest that the ubiquitination level plays an important role in microglial activation, and focusing on UPP degradation of inflammatory proteins may have potential therapeutic value in treating PD.

The specific inhibitor of HSP90, 17‐AAG, is a mature product that is already in phase III clinical trials for cancer treatment (Pillai et al., 2020). Recent studies reported that the protein quality control function of HSP90 participates extensively in PD pathogenesis (Pratt et al., 2015), as multiple pathological proteins involved in PD, including α‐synuclein, are HSP90 clients (Burmann et al., 2020). The increased HSP90 expression in PD is positively correlated with α‐synuclein aggregation, and inhibition of HSP90 attenuates NLRP3 inflammasome activation (Nizami et al., 2021; Uryu et al., 2006). Our results revealed that NLRP3 also acts as an HSP90 client and further support the value of HSP90 inhibition in treating PD at the anti‐inflammatory level; they also highlight the importance of molecular chaperones in regulating Parkin function and PD pathogenesis. However, the effects of HSP90 inhibitors in PD remain controversial, with some studies showing that HSP90 inhibition could *accelerate* α‐synuclein aggregation (Bohush et al., 2019). More investigations are needed to evaluate the functions of two HSP90 isoforms, HSP90α and HSP90β, in PD pathogenesis.

This research has some major limitations. First, the mouse model we used knocked out Parkin in all kinds of cells and not in microglia conditionally, and we therefore could not rule out the involvement of other nerve cells in this process. Second, we did not induce Parkin overexpression in microglia of Parkin KO mice; therefore, further studies should explore the therapeutic role of Parkin supplementation. Third, an inhibition of MCC950 should be used in vivo to certify causal relationship between NLRP3 inflammasome activation and neurodegeneration. Finally, the HSP90 inhibition experiments were only performed in vitro, and in vivo investigations are required for confirmation.

## 5 CONCLUSION

Ultimately, our study revealed that Parkin regulates microglial NLRP3 degradation and protects neurons by mediating microglial activation.

## AUTHOR CONTRIBUTIONS

Design: Yi‐Qun Yan, Jia‐Li Pu. Execution: Yi‐Qun Yan, Ran Zheng, Yi Liu, Yang Ruan, Zhi‐Hao Lin, Nai‐Jia Xue, Ying Chen. Analysis: Yi‐qun Yan. Writing: Yi‐qun Yan, Ran Zheng. Editing of final version of the manuscript: Jia‐li Pu, Bao‐Rong Zhang.

## FUNDING INFORMATION

This study was supported by the National Natural Science Foundation of China (No. 82271268, No. 82271444, and No. 82001346), and the Key Research and Development Program of Zhejiang Province (No. 2020C03020).

## CONFLICT OF INTEREST STATEMENT

All authors claim that there are no conflicts of interest.

## D ATA AVAILABILITY STATEMENT

The datasets used and/or analyzed during the current study are available from the corresponding author on reasonable request.

## Supporting information


Figure S1.

Figure S2.

Figure S3.

Figure S4.

Figure S5.
