# Supplementary figures and images for: Parkin regulates microglial NLRP3 and represses neurodegeneration in Parkinson's disease
Source: Aging Cell. 2023 Apr 7;22(6):e13834. doi: 10.1111/acel.13834 (PMC10265164; doi:10.1111/acel.13834)

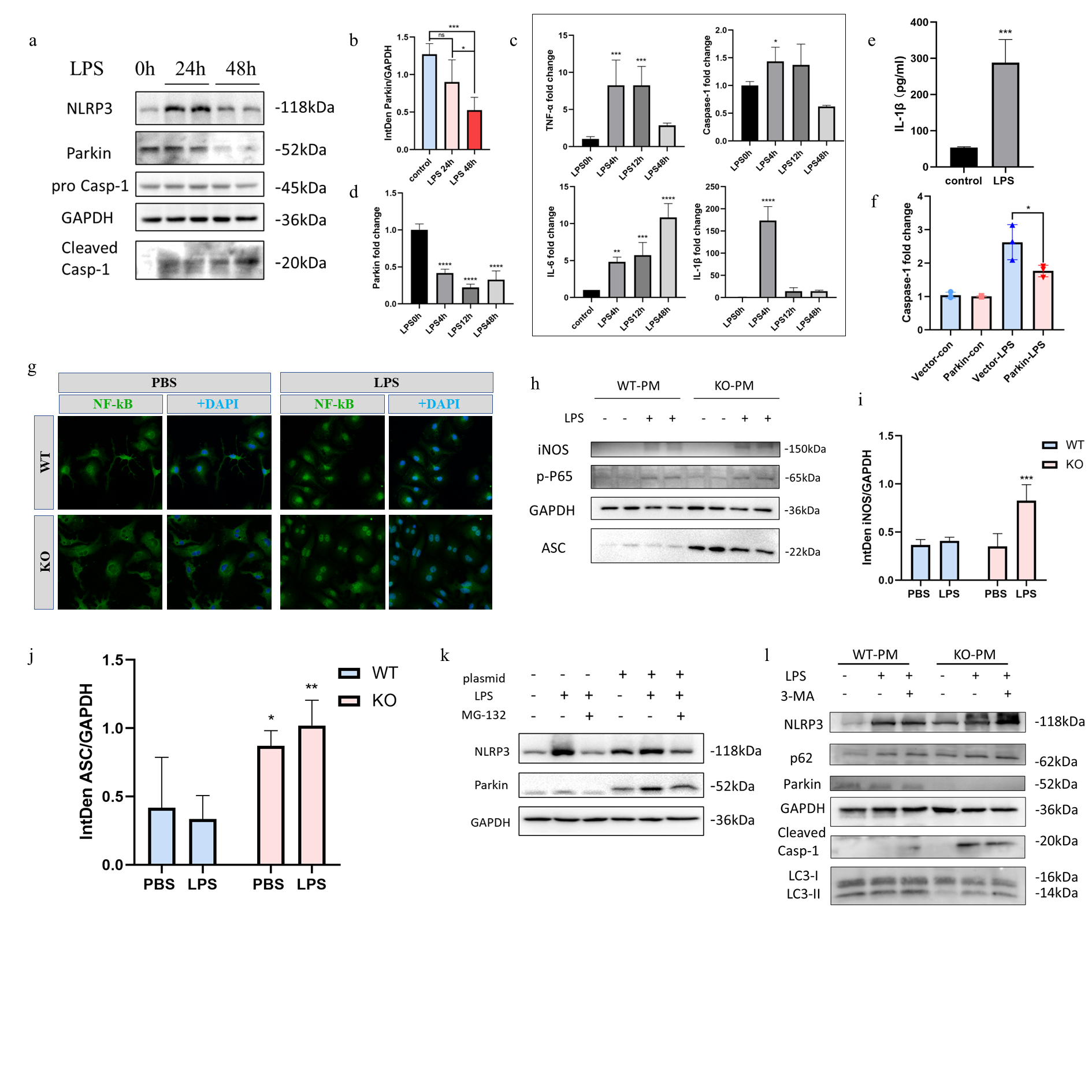

Supplement: Supplementary file 1 — Figure S1. [file ACEL-22-e13834-s004.png]

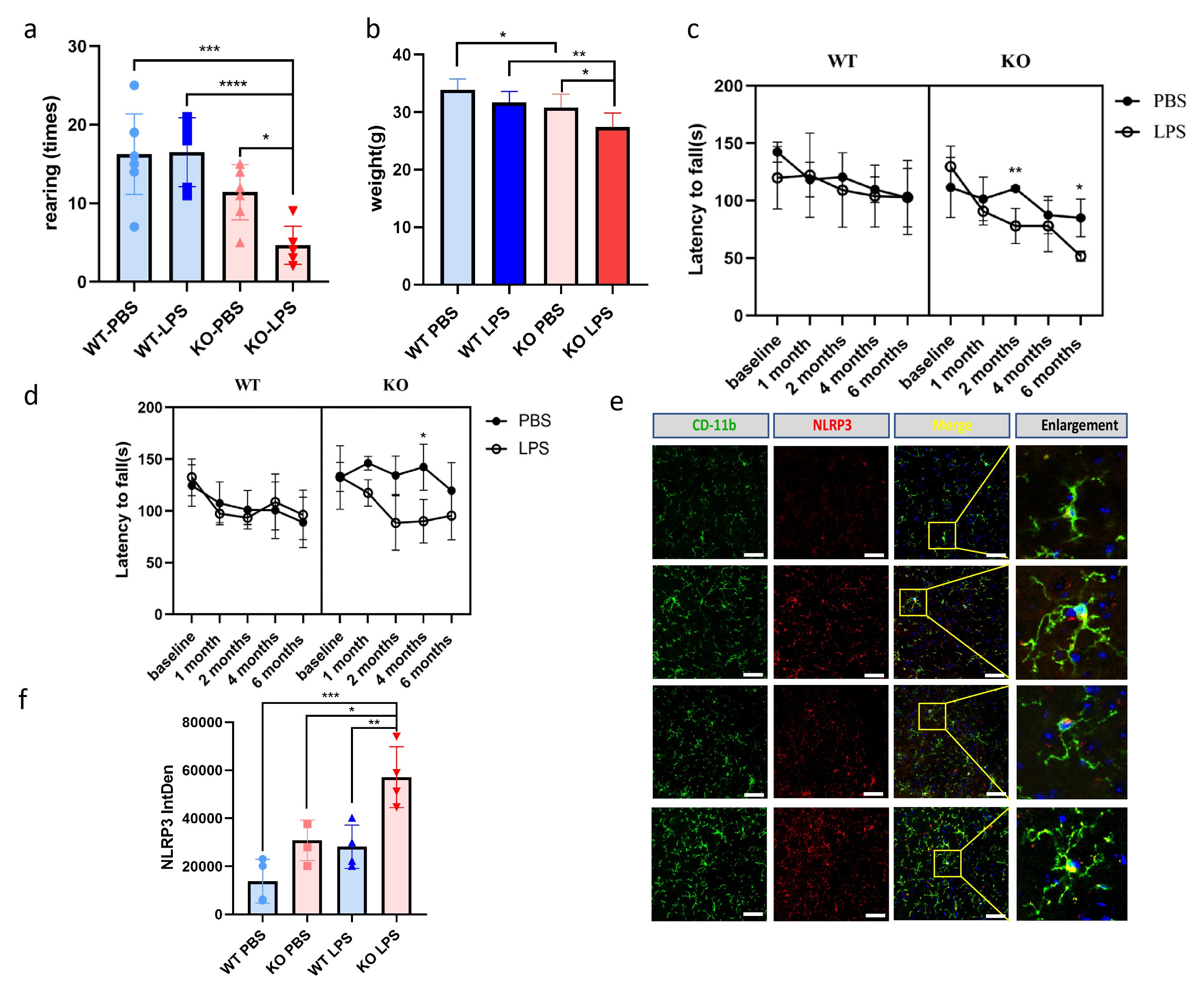

Supplement: Supplementary file 2 — Figure S2. [file ACEL-22-e13834-s005.png]

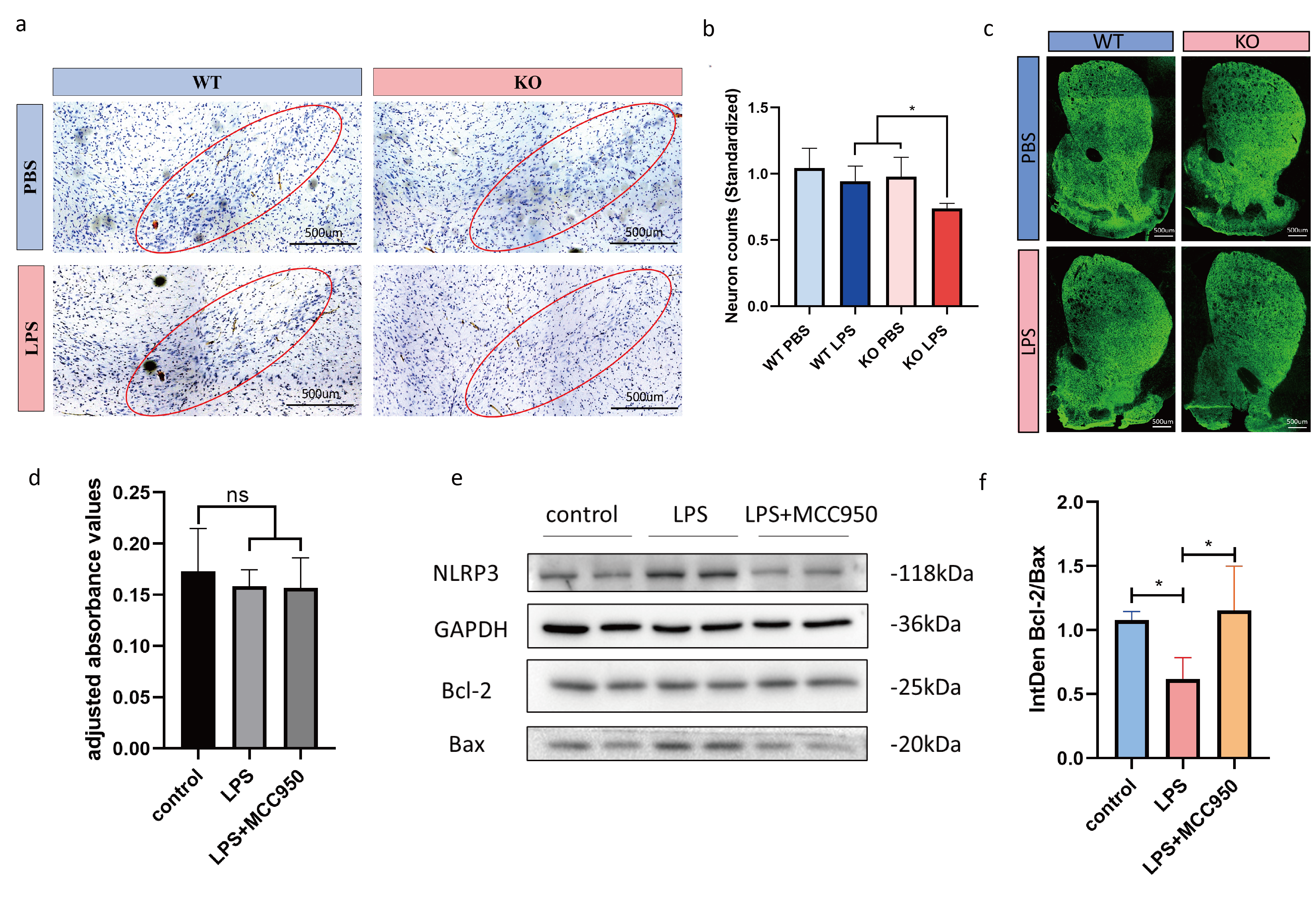

Supplement: Supplementary file 3 — Figure S3. [file ACEL-22-e13834-s002.png]

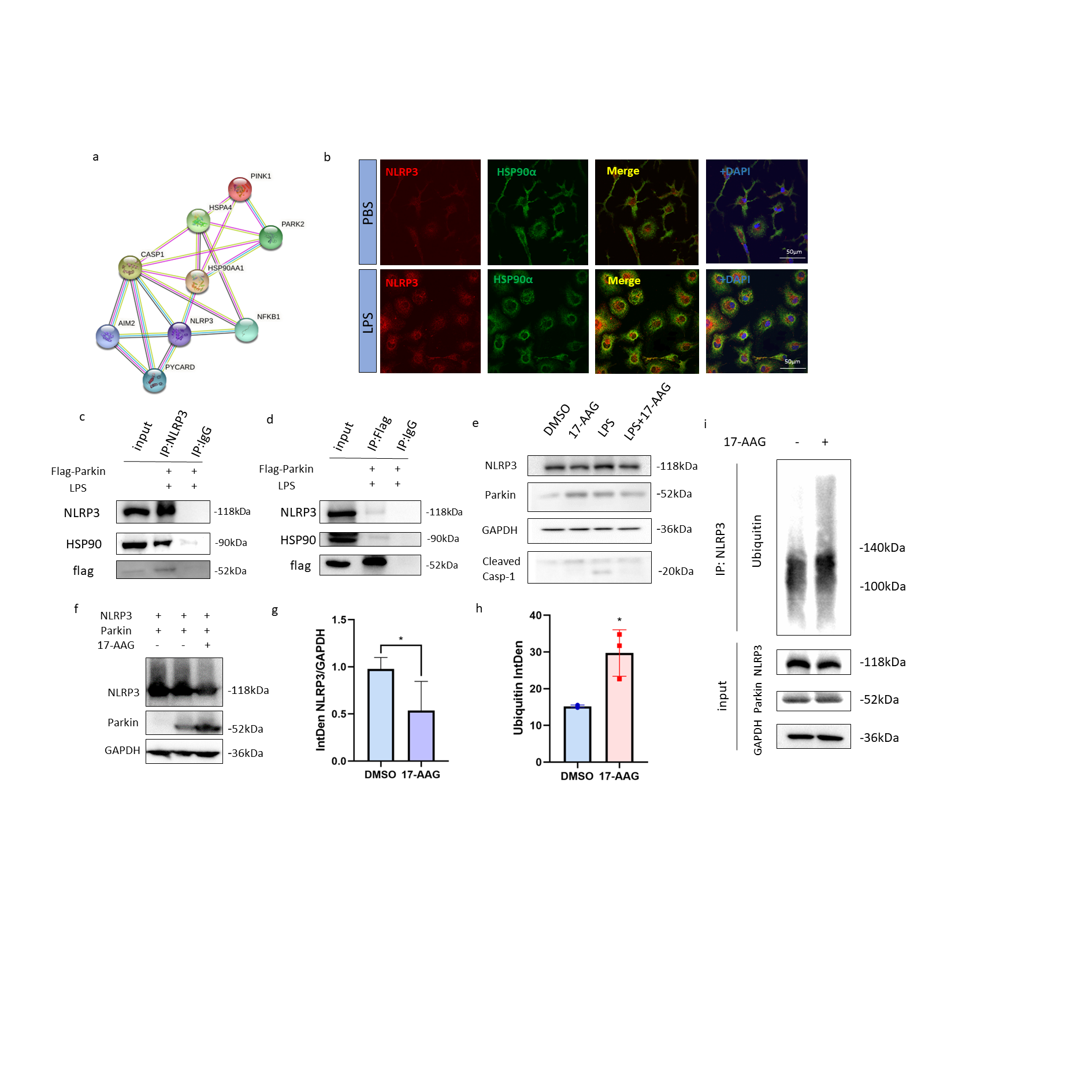

Supplement: Supplementary file 4 — Figure S4. [file ACEL-22-e13834-s001.png]

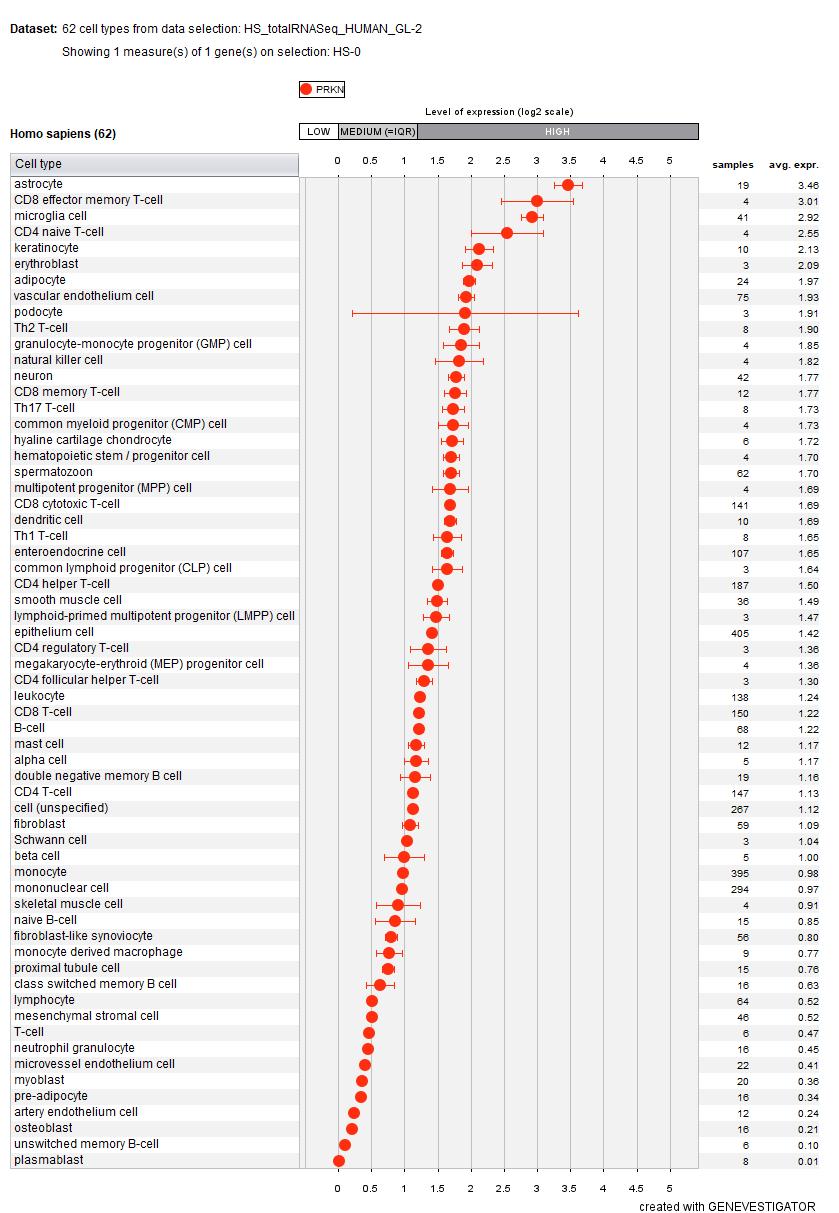

Supplement: Supplementary file 5 — Figure S5. [file ACEL-22-e13834-s003.jpg]
